# Computed tomography in the diagnosis of bilateral renal tuberculosis: diagnostic value, limitations, and future directions

**DOI:** 10.3389/fmedt.2025.1592592

**Published:** 2025-08-29

**Authors:** Zhong Tian, Cheng Zhu, Neng Zhang, Bo Yu, Ni Fu

**Affiliations:** ^1^ Department of Urology, The Second Affiliated Hospital of Zunyi Medical University, Zunyi, China; ^2^ Department of Urology, The Affiliated Hospital of Zunyi Medical University, Zunyi, China

**Keywords:** CT, tuberculosis, bilateral renal tuberculosis, imaging examination, diagnosis

## Abstract

As equipment improves and technology advances, the application of Computed Tomography (CT) in clinical disease diagnosis has become increasingly widespread, particularly demonstrating significant advantages in diagnosing solid lesions. However, CT scans still face challenges, including insufficient sensitivity and an inability to assess renal function when diagnosing bilateral renal tuberculosis (BRTB). By reviewing relevant high-quality literature, we compared the sensitivity, specificity, advantages, and limitations of USG, KUB, IVU, MRI, PET-CT, and CT in the diagnosis of BRTB. CT offers higher clinical detection rates and reduces the economic burden on patients compared to other imaging methods, making it the preferred modality for imaging in patients with BRTB. AI-assisted diagnosis and the integration of CT with PET may represent promising future directions for CT imaging.

## Introduction

Currently, tuberculosis (TB) remains one of the deadliest infectious diseases worldwide (1, 2). Urogenital TB accounts for 30%–40% (3) of extrapulmonary TB cases and is the second most common form of extrapulmonary TB (4). Renal TB is the most common form of urogenital TB (3, 5), with unilateral renal TB accounting for more than 90% of cases, while BRTB makes up less than 10%. BRTB is a chronic, progressive infectious disease caused by Mycobacterium TB, either through hematogenous spread (3, 6, 7) or via the reflux of infected urine from one kidney, leading to bladder fibrosis and contracture, which subsequently affects the contralateral kidney (8). BRTB can lead to severe renal damage, and renal function may not be able to compensate. Due to its insidious onset and the absence of specific clinical manifestations, early diagnosis of BRTB is challenging (9, 10).

CT imaging is a common diagnostic tool for renal diseases. It can reveal intrarenal hypodense foci, hydronephrosis, renal scarring, calcification, renal atrophy, and bladder contracture in patients with BRTB. CT provides more detailed pathological and anatomical information and is superior to IVU and USG in assessing the extent of renal lesions (10). Therefore, CT plays a crucial role in diagnosing BRTB and assessing its severity (11).

Although fees for various imaging examinations vary by country and region, as well as depending on health insurance coverage and investment levels, the cost of CT scans has decreased in many areas due to advancements in equipment and technology (12). China, one of the countries with the highest incidence of TB, has seen the price of plain CT scans drop from 240 RMB per area to 200 RMB, while enhanced scans have decreased from 285 RMB to 240 RMB, with further reductions expected.

We used the PUBMED database to retrieve literature on “Renal TB” and “Imaging examination” for review, aiming to encourage clinicians to be more vigilant about BRTB, so that it can be diagnosed earlier and treated promptly to protect renal function.

## Imaging studies

Imaging studies are generally considered suggestive rather than definitive for confirming or excluding BRTB. Based on the patient's history, laboratory findings, and CT imaging suggesting BRTB, the clinician can confidently diagnose the condition (13). There is a correlation between the time to diagnosis and the severity of BRTB (14), with radiographic findings reflecting the extent of renal lesions. Each imaging modality has its advantages, and mastering their features aids in early diagnosis and timely treatment, thereby reducing the incidence of BRTB (13, 15).

## USG

Ultrasonography (USG) is a well-established imaging technique for detecting morphological abnormalities in kidney TB, offering advantages such as being non-invasive, dynamic, economical, and convenient (13, 16, 17). Radiographic findings of renal TB may include calcifications, hydronephrosis, parenchymal masses, cavities, dilated or constricted renal pelvis, renal pus accumulation, and renal atrophy (18–21). When a definitive clinical diagnosis cannot be made and renal lesions need to be identified, diagnosis can be confirmed through ultrasound-guided percutaneous renal biopsy with pathological examination (22).

The disadvantages of USG include limited sensitivity and accuracy (23), as well as an inability to assess the extent of TB spread or evaluate renal function (24, 25). USG may lack sensitivity to calcifications. In cases of diffuse renal involvement without additional imaging findings, B-mode ultrasound may fail to detect bilateral renal abnormalities, leading to missed diagnoses (26).

## KUB

More than 90% of renal TB originates from disseminated pulmonary TB, and approximately 50% of patients have chest radiographs that appear negative (27). The most common imaging findings on x-ray for BRTB are calcifications or renal scarring. Early calcifications in the renal parenchyma are typically granular or curvilinear (5, 28), followed by the formation of a granulomatous mass, which appears spherical or nodular (27). Calcification is rare in the early stages of the disease, with a detection rate of only 24%–44% (27).

The detection rate of TB with flat urinary tablets is low, hindering early diagnosis. CT can detect fine calcifications (26) that are not visible on x-ray and offers clear advantages over KUB in terms of detection and accuracy.

## IVU

Intravenous urography (IVU) is a key examination for detecting anatomical and functional changes in the kidneys and is considered the gold standard for diagnosing urogenital TB (13, 29). IVU examination can reveal early signs of BRTB, including calyx erosion, dilation, and loss of calyx sharpness. The renal contour becomes irregular, blurred, and rough as the disease progresses, eventually showing a “plumage” or “moth-eaten” appearance (30). In the late stages, may show extensive cavities (13), fibrous stenosis, cortical scarring, calcification, abscesses, and fistula formation. Compared to USG, CT, and MRI, IVU's high spatial resolution can detect subtle erosive changes in the urothelium (31).

IVU has a miss rate of 10%–15% (24) and may have difficulty distinguishing between hydronephrosis and TB granuloma. The utility of IVU is closely linked to the degree of renal impairment on the affected side. In most patients with BRTB, renal function is severely compromised in the middle and late stages, and the kidney with severe lesions may exhibit poor or absent development (32). As a result, the significance of this examination is limited in these patients. Retrograde pyelography is typically used when IVU results are unsatisfactory or when no contrast is excreted by the affected kidney. Advancements in CT technology have gradually replaced IVU with CT urography. Unlike IVU, CT can easily differentiate between TB granuloma and hydronephrosis.

## MRI

MRI can clearly display renal morphological details and visualize the ureter (29). MRI is considered superior to CT (33) for detecting and assessing TB. MRI has high sensitivity and accuracy in diagnosing BRTB, especially when compared to CT or USG (29, 34). MR urography (MRU) can better display the upper urinary tract in cases of a dilated bladder (35), but it is less useful in determining the site of stenosis due to impaired renal function (36, 37). MRI plays a key role in diagnosing and evaluating intracranial TB (38).

In some cases, MRI may be more accurate than CT. CT cannot detect TB lesions that are 0.5–2.0 mm in size, while MRI will show a low signal (33). When CT does not suggest a TB lesion, MRI may assist clinicians in identifying BRTB.

MRI helps distinguish large TB lesions from other mass lesions. Enhanced MRI reveals local tissue edema and vasoconstriction from active inflammation, leading to focal hypoperfusion that can be challenging to distinguish from acute pyelonephritis of other etiologies (39, 40). Additionally, MRI is more expensive and has longer wait times compared to other common imaging methods.

## PET-CT

PET-CT plays a crucial role in diagnosing extrapulmonary TB, including urinary tract TB (41), and helps differentiate between benign and malignant tumors. It has 95% specificity and 83% sensitivity (42), and provides functional data on metabolism, drug penetration, and immune control, significantly aiding drug development and protocol selection (43).

PET-CT has limitations after radionuclide injection, as it cannot distinguish renal TB from conditions such as acute and chronic glomerulonephritis, pyelonephritis, acute tubular degeneration, and necrosis caused by toxic substances (44). As a result, it cannot definitively diagnose renal TB, and its high cost is another drawback.

## CT

CT offers the advantage of high spatial resolution and no overlapping anatomical structures, making it crucial in diagnosing BRTB (40). CT can effectively identify pathological changes and disease progression in patients with BRTB. Examination of patients with BRTB may reveal renal morphological changes, low-density foci in the renal parenchyma, calcification, hydronephrosis, bladder contracture, renal dysfunction, and damage. It can also sensitively detect abscesses and ureteral stenosis (45, 46).

CT is the most sensitive method for identifying intrarenal calcification, offering high accuracy, precision, and sensitivity (25, 47, 48). CT provides more detailed pathological and anatomical information and offers significant advantages over IVU, retrograde pyelography (RGP), and USG in detecting multiple small urothelial lesions (40).

CT does not require bowel preparation, unlike IVU, and directly visualizes renal parenchyma (40) without assessing renal function.

Multi-detector computed tomography (MDCT) allows dynamic evaluation of the kidneys at different stages of contrast, helping to assess the extent of renal lesions, obstruction, and associated complications (19).

CT urography (CTU) can reveal early imaging manifestations of renal TB, which may also be detected by IVU. It has high diagnostic value, with sensitivity up to 90% and specificity up to 85% (49). CTU provides high-resolution imaging using MPR and CPR techniques, with post-processing technology to assess kidney, ureter, and bladder lesions, as well as the degree of surrounding tissue invasion (50). This helps clinicians make accurate diagnostic and qualitative assessments, offering clear advantages in diagnosing complex BRTB and planning surgical treatment.

When diagnosing kidney diseases, the radiation dose from CT is relatively low (51). For most individuals, CT scans do not significantly increase the risk of cancer. Although CT scans expose patients to some radiation, the risks can be minimized with proper use and protective measures. The medical benefits of CT scans for patients with BRTB outweigh any potential long-term risks (52).

## Discussion

Risk factors for BRTB include rural residence, urinary calculi, low BMI, and previous use of ureteroscopy (7). The disease progresses through different stages (40, 53). In the early stage, it is primarily characterized by destruction of the renal papilla and necrosis of granulomas in the renal parenchyma. As the disease progresses, low-density and calcified lesions develop. In the later stages, hydronephrosis, renal atrophy, and renal reabsorption occur due to fibrosis-induced stenosis.

**Figure 1:**
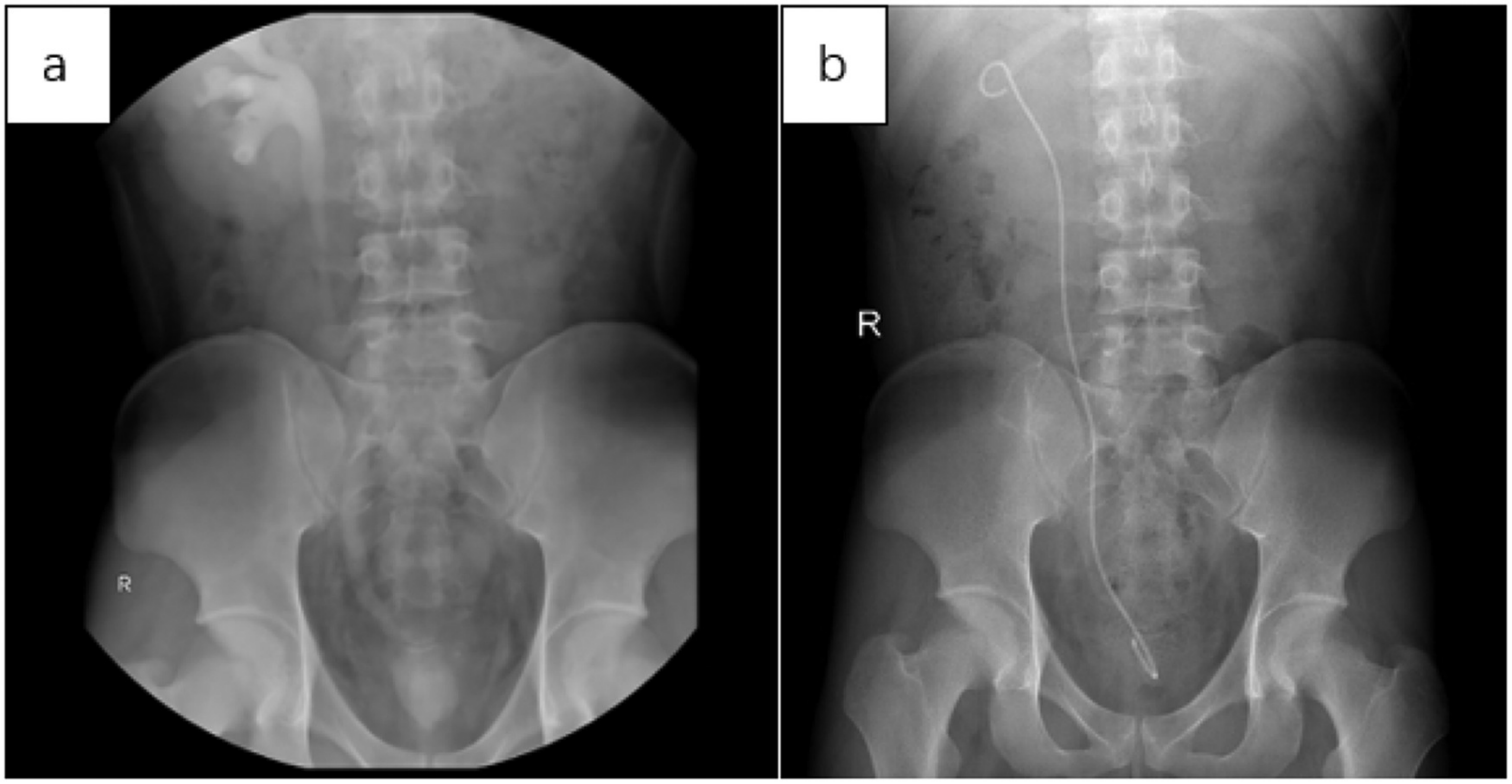
**(a)** in the IVU, mild expansion was observed in the right calyx, renal pelvis, and upper segment of the ureter. There was no contrast in the left calyx, ureter, and renal pelvis, with contrast filling in the bladder, which showed significantly reduced volume. This suggests that the left kidney is non-functional and there is bladder contracture. **(b)** Three months after implantation of the right ureteral stent, KUB imaging showed a double “J” tube shadow in the right renal pelvis, ureter, and bladder. The position of the tube was normal, with the tip located in the right renal pelvis, and no obvious abnormalities were noted.

**Figure 2:**
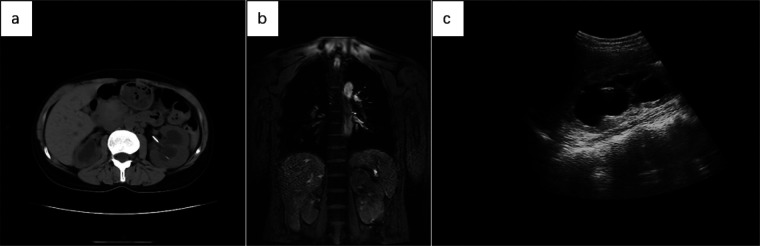
**(a)** abdominal CT showed increased volume in both kidneys, with more pronounced enlargement of the left kidney and migration of the left renal pelvis following ureteral stent placement. **(b)** The coronal MRI T2WI of the chest and upper abdomen showed irregular morphology, increased volume, and prominent findings in the left kidney. **(c)** USG revealed significant expansion of the left renal pelvis, with hypoechoic areas and a thinning renal cortex.

**Figure 3:**
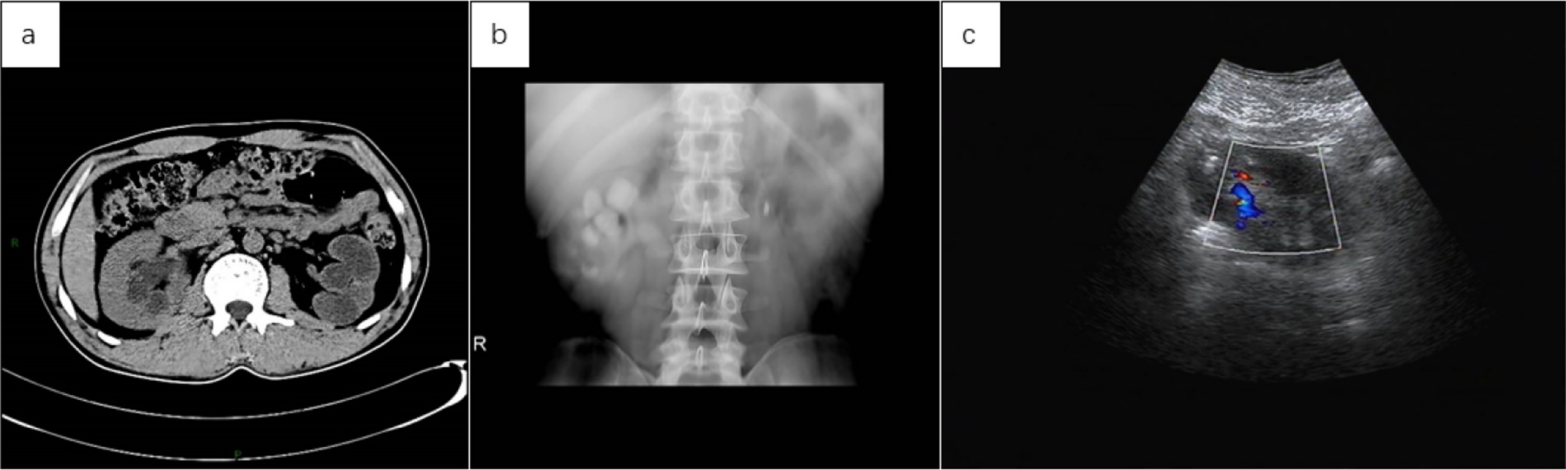
**(a)** the abdominal CT scan revealed a reduced left kidney with significant dilation of the renal pelvis and thinning of the renal cortex, suggesting a non-functional left kidney due to pus. The right kidney appears compensatorily enlarged, with slight dilation of the right renal pelvis. **(b)** Following reexamination after left nephrectomy, IVU revealed slight dilation of the right calyx and renal pelvis, no contrast filling in the left kidney, and a strip-like high-density shadow in the left hilar region, suggestive of a surgical stapler shadow. **(c)** Repeat examination after left nephrectomy showed no obvious abnormalities in the size of the right kidney, with slight dilation of the renal pelvis on USG.

**Figure 4:**
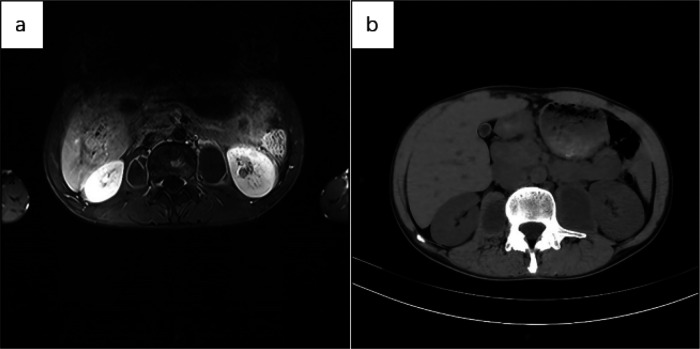
**(a)** axial MRI of the upper abdomen (T1WI with lipid suppression) showed a patchy hyperintense signal in the lumbar vertebral body and bilateral paravertebral capsules. The cystic walls were smooth, with uniform thickness, and the contents within the capsule exhibited a consistent low signal. Given the medical history, spinal TB with a paravertebral cold abscess was considered. The left kidney showed a small, non-enhancing low-signal area, suggestive of a cystic lesion. **(b)** Axial CT of the upper abdomen showed no obvious abnormalities in the morphology, size, or density of both kidneys. Bilateral cystic low-density shadows were seen beside the lumbar vertebral body, with uniform wall thickness and no wall nodules. The sacs exhibited a uniform low-density shadow, and no septation was noted.

**Figure 5:**
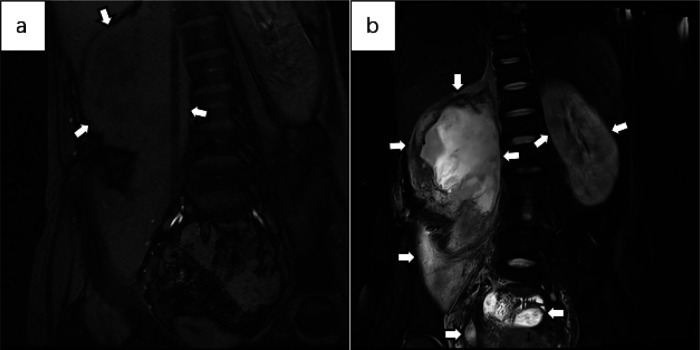
**(a)** the abdominal MRI T1WI fat-saturated coronal image shows an irregular shape of the right kidney, with the renal cortex noticeably thickened and uneven. The renal calyx and pelvis appear with slightly lower signal intensity. The lesion extends to the perirenal area and the psoas major muscle, suggesting the formation of a psoas major abscess. There is no obvious abnormality in the shape or size of the left kidney. **(b)** The MRI T2WI fat-suppressed image in the coronal view shows patchy high signal areas in the perirenal region and renal pelvis on the right side, suggesting TB pyonephrosis with the formation of a psoas abscess. The left kidney is slightly enlarged, with possible edema. Low signal areas are seen in the renal parenchyma, consistent with early signs of renal TB. Abnormal high signal areas are observed in the pelvis, which, combined with the patient's medical history, suggest pelvic TB.

**Figure 6:**
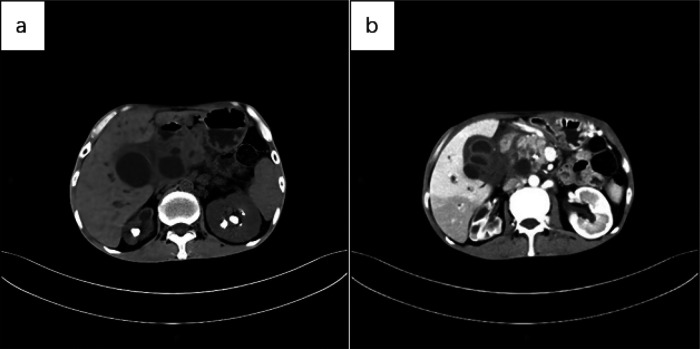
**(a)** CT scan of the abdomen showed multiple nodular high-density shadows in both kidneys, with significant reduction in the volume of the right kidney and uneven thickening of the renal cortex, suggestive of renal self-resection. The left kidney exhibited compensatory enlargement. Additionally, multiple cystic mass shadows were observed in the upper abdomen. **(b)** Abdominal CT (cortical phase) revealed uneven enhancement of the right kidney, with multiple low-density areas and increased volume of the left kidney. No significant enhancement was observed in the sac of the upper abdominal lesion, and the cystic wall showed uniform enhancement.

**Figure 7:**
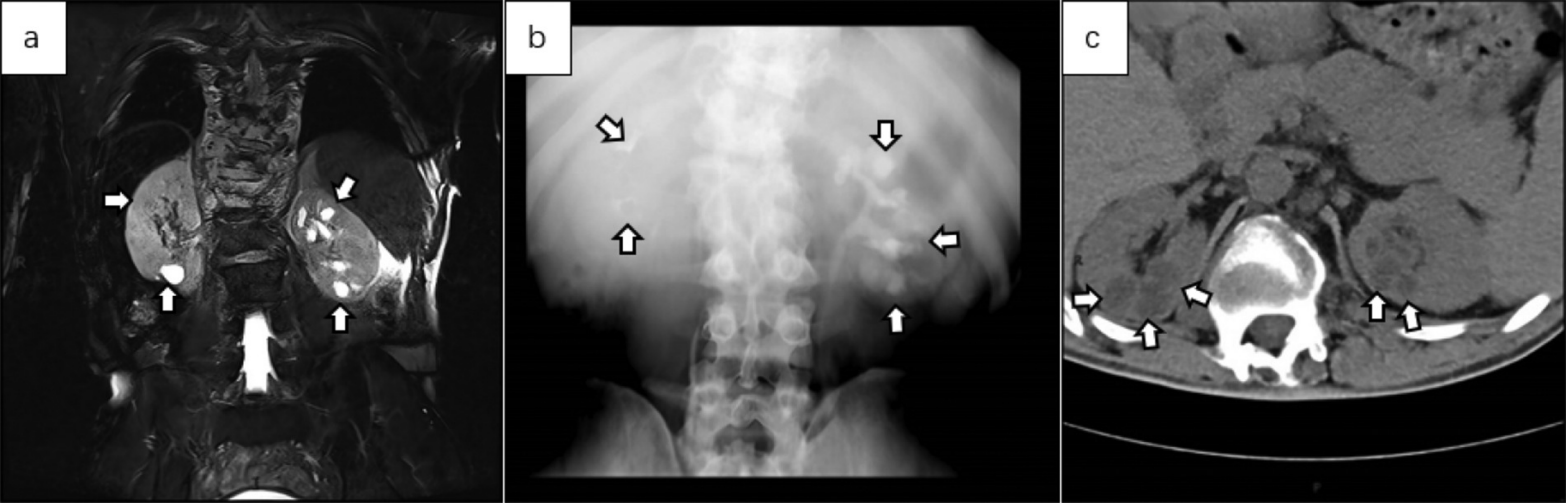
**(a)** coronal abdominal MRI T2WI showed multiple cystic high-signal areas in both kidneys, predominantly in the left kidney. The capsule exhibited a uniform signal, and no obvious morphological abnormalities were noted in either kidney. **(b)** IVU revealed contrast filling of the renal capsule in the left kidney, with expansion of the left calyx, poor contrast visualization of the right calyx and renal pelvis, and normal development of both ureters. **(c)** Plain axial CT images of the abdomen showed no obvious abnormalities in the morphology of either kidney, slight dilation of the upper segments of both ureters, and multiple cystic low-density shadows in the renal parenchyma.

Below are imaging results from seven patients with BRTB. IVU is more sensitive than KUB for detecting abnormal kidney morphology and size. It can also diagnose renal function and bladder contracture based on contrast agent filling (Figure 1). Compared to USG, CT revealed abnormal bilateral kidney morphology and size (MRI results were similar to CT), providing a clearer view of hydronephrosis (Figure 2). IVU is more sensitive than USG in detecting calyx and renal pelvis expansion (hydronephrosis), but it is less effective in showing the extent of renal lesions and parenchyma. CT, on the other hand, can distinguish lesions from renal parenchyma, providing high sensitivity for hydronephrosis and low-density lesions (Figure 3). CT and MRI were comparable in detecting lesions, but MRI was more sensitive in diagnosing soft tissue lesions and distinguishing them from normal tissue (Figure 4). The two MRI sequences showed no significant difference in detecting renal TB foci, but T2-weighted images (T2WI) were clearer in distinguishing lesions from normal tissue compared to T1-weighted images (T1WI) (Figure 5). CT examination is sensitive in detecting abnormal renal lesions, morphology, and size. Enhanced CT can assess renal blood flow to evaluate renal function and allow for bilateral comparison (Figure 6). CT and MRI provide a clearer and more intuitive assessment of kidney morphology and size compared to IVU. Additionally, CT is more sensitive in detecting hydronephrosis and ureteral dilatation (Figure 7). In conclusion, CT is effective in identifying TB foci, abnormal kidney morphology and size, hydronephrosis, and assessing renal function.

Some patients with BRTB may have one or more large nodules in the kidneys, which appear as lesions of varying sizes with clear boundaries on cross-sectional images. Enhanced CT may show peripheral enhancement of the nodules, necessitating differentiation from renal cancer.

There is a partial overlap in the imaging features of kidney TB and both benign and malignant kidney tumors (54), which contributes to clinical delays in diagnosis. Despite significant advances in CT and its high differential diagnostic value, qualitative diagnosis remains challenging (55). Ultrasound, CT, and MRI have high sensitivity for detecting renal lesions, but none can provide an accurate and reliable qualitative diagnosis. When clinical diagnosis is challenging and a qualitative assessment is needed, renal biopsy (56) should be considered. Over the past few decades, needle biopsy and pathological examination have been the primary methods for diagnosis. However, they carry a risk of disseminated metastasis (54) and can increase patient burden and pain. One aim of our review on CT-assisted diagnosis of BRTB is to provide readers with guidance on using CT to aid diagnosis, thereby reducing the need for renal biopsies and the risk of infection dissemination.

We compared the sensitivity, specificity, advantages, and limitations of USG, KUB, IVU, MRI, PET-CT, and CT (Table 1) (33, 49). IVU was previously the gold standard for diagnosing renal TB, but it often failed to reveal any signs, or showed no signs when kidney lesions were severe, leading to its gradual replacement by CT. PET-CT offers the highest sensitivity for diagnosing renal TB, but due to its inability to distinguish between other inflammatory diseases and its high cost, it is not the preferred imaging modality for BRTB. MRI has comparable sensitivity and specificity to CT, but its time-consuming and expensive nature makes it less convenient and less widely used in clinical settings compared to CT. By summarizing the diagnostic performance, advantages, and limitations of these imaging techniques, this article emphasizes that CT can be considered the preferred imaging modality for BRTB.

**Table 1:** By comparing the sensitivity, specificity, advantages, and limitations of USG, KUB, IVU, MRI, PET-CT, and CT in diagnosing BRTB, we found that CT offers a higher clinical detection rate and reduces the economic burden on patients compared to other imaging modalities.

Category	Sensitivity	Specificity	Advantages	Limitations
USG	59%	More than 86%.	Convenient	The sensitivity is low, preventing the determination of the extent of TB lesion spread and the evaluation of renal function.
KUB	30%–40%	More than 90%.	Sensitive to calcification	It is not sensitive to microcalcification, and 50% of patients had negative chest x-ray results.
IVU	88%	More than 90%.	It is one of the most useful tests for detecting anatomical and functional changes in the kidney, as it reveals subtle erosive changes in the urothelium.	There is a 10–15% misdiagnosis rate for active renal TB, and it is difficult to differentiate between hydronephrosis and TB granuloma. Poor imaging or even a lack of imaging may occur.
MRI	More than 90%.	More than 90%.	No radiation exposure and high soft tissue contrast.	The procedure is time-consuming and more expensive.
PET-CT	83%–98%	95%	It can assess renal function and differentiate between benign and malignant tumors.	It cannot identify renal insufficiency caused by BRTB or other kidney diseases, and it is costly.
CT	90%–95%	More than 85%.	The anatomical structure is distinct, with high density and spatial resolution. It is highly sensitive in identifying hydronephrosis, intrarenal calcification, and low kidney density, offering high accuracy, precision, and sensitivity.	MRI is less sensitive to mild calyceal deformities and renal parenchymal edema than CT, and its interpretation needs to be combined with the patient's clinical background (history and laboratory test results).

The insufficient sensitivity of CT scans limits their ability to diagnose BRTB at an early stage, which is their greatest drawback. Furthermore, CT scans cannot accurately assess renal function and cannot directly guide preoperative evaluation or determine the timing of surgery for clinicians. Looking ahead, the integration of CT with other imaging techniques or the application of artificial intelligence (AI) in clinical settings may significantly help address these limitations.

## Conclusion and future prospects

The onset of BRTB is insidious, with no specific clinical manifestations, making clinical diagnosis challenging. This review summarizes the advantages and disadvantages of common clinical imaging techniques and highlights that CT offers a high detection rate and reduces economic burden on patients, making it the preferred imaging modality for those with BRTB.

The challenge of CT examination lies in detecting early morphological and functional changes in renal TB and distinguishing it from benign and malignant kidney tumors. With TB can be detected and diagnosed earlier in the future, thereby reducing related complications and improving patients' quality of life. It is also hoped that, in the future, BRTB and benign and malignant renal tumors can be clearly identified without the need for renal biopsy.

AI has recently garnered significant attention due to its widespread use in healthcare applications. Using AI to extract and enhance information from medical images represents a major breakthrough in the field of medical imaging (57). In future developments, the integration of AI with CT may offer an effective solution to the challenge of “CT failing to diagnose early-stage BRTB”.

Radionuclide imaging techniques have been applied to various aspects of TB diagnosis, including evaluating lesion characteristics, assessing treatment efficacy, predicting recurrence, and conducting pharmacokinetic studies of new anti-TB drugs (44, 58). The integration of PET-CT and CT is also one of the promising future methods for diagnosing BRTB, addressing issues such as “inability to assess renal function” and “differentiating renal TB from other infectious lesions”. We look forward to large-scale prospective studies in this field, which will undoubtedly drive further development in related areas or disciplines.
